# *AR* (CAG)_n_ Microsatellite and *APEX1* c.444T>G (p.Asp148Glu) Polymorphisms as Independent Prognostic Biomarkers in Prostate Cancer: Insights from an Argentinian Cohort

**DOI:** 10.3390/cancers16223815

**Published:** 2024-11-13

**Authors:** Gaston Pascual, Agustina Sabater, Juan Bizzotto, Rocio Seniuk, Pablo Sanchis, Sabrina Ledesma-Bazan, Estefania Labanca, Carlos Scorticati, Osvaldo Mazza, Elba Vazquez, Ayelen Toro, Federico Prada, Geraldine Gueron, Javier Cotignola

**Affiliations:** 1 Departamento de Química Biológica, Facultad de Ciencias Exactas y Naturales, Universidad de Buenos Aires, Buenos Aires C1428EGA, Argentina; gpascual@qb.fcen.uba.ar (G.P.); asabater@qb.fcen.uba.ar (A.S.);; 2 Instituto de Química Biológica de la Facultad de Ciencias Exactas y Naturales (IQUIBICEN), CONICET-Universidad de Buenos Aires, Buenos Aires C1428EGA, Argentina; 3 Instituto de Tecnología (INTEC), Universidad Argentina de la Empresa (UADE), Buenos Aires C1073AAO, Argentina; 4 Department of Genitourinary Medical Oncology and The David H. Koch Center for Applied Research of Genitourinary Cancers, The University of Texas MD Anderson Cancer Center, Houston, TX 77030, USA; 5 Departamento de Urología, Ciudad Autónoma de Buenos Aires (CABA), Hospital de Clínicas José de San Martín, Universidad de Buenos Aires, Buenos Aires C1120AAF, Argentina

**Keywords:** prostate cancer, polymorphisms, androgen receptor, APEX1, biochemical relapse, genetic biomarkers

## Abstract

Background/Objectives: Prostate cancer (PCa) is the leading malignancy and the third most common cause of cancer-related death in Argentinian men. Predicting outcomes in localized PCa remains difficult due to tumor heterogeneity. In this study, we assessed the impact of *AR* (CAG)_n_ and *APEX1* c.444T>G polymorphisms on biochemical relapse in Argentine patients with localized PCa. Methods: We genotyped blood samples from 123 PCa patients for *AR* (CAG)_n_ and *APEX1* p.Asp148Glu (c.444T>G) polymorphisms. Associations with clinicopathological parameters and biochemical relapse-free survival (BRFS) were assessed. Results: *AR* (CAG)_20–23_ was associated with a family history of breast/ovarian cancer (*p* = 0.0469). The combination of *AR* (CAG)_20–23_ and *APEX1* c.444TT/GG correlated with a 2.89 times higher risk of biochemical relapse (log-rank *p* = 0.006). Multivariable analysis confirmed *AR* and *APEX1* polymorphisms as independent predictors of biochemical relapse (HR = 3.95, *p* = 0.002). In patients with PSA levels <10 ng/mL, combined *AR* (CAG)_20–23_ and *APEX1* c.444TT/GG genotypes were significantly associated with an increased risk of biochemical relapse (HR = 2.61, *p* = 0.044). Multivariable analysis confirmed the prognostic significance of these genotypes (HR = 3.44, *p* = 0.02). Conclusions: This study has identified *AR* (CAG)_n_ and *APEX1* c.444T>G polymorphisms as independent predictors of PCa relapse in Argentinian patients, suggesting their potential use in improving prognostic models.

## Simple Summary

This study explores the association of two gene polymorphisms (*AR* (CAG)_n_ and *APEX1* c.444T>G (p.Asp148Glu)) with biochemical recurrence in Argentinian patients with prostate cancer. The findings suggest that patients with certain alleles (*AR* (CAG)_M_ and the heterozygous *APEX1* c.444TG genotype) are linked to an increased risk of prostate cancer relapse, even when accounting for conventional clinicopathological variables. Notably, patients with a combined *AR* (CAG)_M_ and *APEX1* c.444TT/GG genotype had the highest risk of relapse, indicating the potential of these polymorphisms as independent predictors. These results are particularly relevant for patients with prostate specific antigen (PSA) serum levels <10 ng/mL, where traditional markers may not predict outcomes. By incorporating these genetic markers into clinical models, this research offers a novel approach to enhance the precision of prostate cancer prognosis and improve patient stratification, ultimately guiding more personalized treatment strategies for high-risk individuals.

## 1. Introduction

Prostate cancer (PCa) is the second most common cancer and the fifth leading cause of cancer-related deaths among men worldwide, with an incidence of 1,467,854 new cases and 397,430 deaths reported in 2022 [1,2]. Despite advances in diagnosis and treatment, the clinical course of PCa remains highly variable, ranging from indolent to aggressive metastatic disease. This variability underscores the need for reliable prognostic biomarkers that can predict disease progression and guide precise treatment strategies [3].

The androgen receptor (AR) is a nuclear receptor that mediates the effects of androgens by binding to androgen response elements (ARE) in the DNA to modulate the transcription of target genes. It regulates the biological processes necessary for normal prostate development and function. In PCa, AR and its signaling pathways are responsible for the growth, proliferation, and survival of tumor cells during the androgen-dependent stage, and activating mutations in *AR* are involved in the development and progression of the disease [4].

The *AR* gene contains a (CAG)_n_ codon repeat polymorphism in the first exon that encodes a polyglutamine (polyQ) tract in the N-terminal transactivation domain. It has been extensively reported that the length of the polyQ tract is inversely correlated with basal and ligand-induced AR transactivation activity [4,5,6,7]. The length of this polyQ tract has also been implicated in various pathologies. In bladder cancer [8], patients with shorter (CAG)_n_ alleles showed significantly decreased 3-year specific relapse-free survival. Studies of breast cancer have shown that shorter (CAG)_n_ repeats in either one or both alleles are positively correlated with histological grade [9]. In PCa, several studies have assessed the relation between the length of this microsatellite and the development of the disease [10]. A meta-analysis of several ethnic groups suggested that short alleles are risk factors for PCa [10]. Nevertheless, these results are inconsistent, as different outcomes have been reported in different populations. Significant associations have been observed in Asian populations [10] and borderline significance has been observed for Caucasian [11] populations, while there have been no significant findings among African populations [10,11]. These reports indicate that the correlation between the length of the (CAG)_n_ microsatellite and clinicopathological features varies from cohort to cohort [12,13], highlighting the importance of population studies and regional approaches. The overall effect of this polymorphism on disease progression remains elusive.

APEX1, a major apurinic/apyrimidinic endonuclease, plays a crucial role in the DNA base excision repair (BER) pathway [14]. APEX1 and other BER enzymes are recruited by androgen response elements to modulate the transcription of AR target genes, a mechanism triggered by DNA damage due to reactive oxygen species [15]. This gene bears a single nucleotide variant (SNV) that causes an amino acid change at residue 148 (c.444T>G; p.Asp148Glu). This variant has been reported to have no impact on *APEX1* endonuclease or DNA binding activities [16]. Interestingly, there are reports associating this polymorphism with an increased risk of developing PCa in a Brazilian cohort [17] and in patients of Asian and mixed descent [18]. On the other hand, other studies have shown that there are no significant associations between this SNV and the risk of PCa in Caucasian and African American men [19], or in patients from Northern India [20,21]. Additionally, some studies have reported that there are no significant associations between PCa and the *APEX1* polymorphism by itself, but the combination of *APEX1* c.444T>G and *XRCC1* p.399R>Q indicates an increased risk of developing PCa in Caucasian men [22].

Given the potential clinical implications of *AR* (CAG)_n_ and *APEX1* c.444T>G (p.Asp148Glu) and their variation in different ethnic groups, it is necessary to explore their prognostic value in other populations. Argentina, with its unique genetic background influenced by European, Amerindian, and African ancestries, provides an ideal setting to study the impact of *AR* and *APEX1* polymorphisms on PCa outcomes [23]. Thus, in this study, we carried out a comprehensive analysis of *AR* (CAG)_n_ and *APEX1* c.444T>G polymorphisms in an Argentinian cohort of 123 PCa patients, studying their associations with clinicopathological parameters and biochemical relapse-free survival (BRFS).

## 2. Materials and Methods

### 2.1. Patients

We designed a hospital-based case study to determine the association between *AR* and *APEX1* polymorphisms and PCa clinicopathological parameters. We retrospectively recruited 123 patients diagnosed with PCa from August 2008 to November 2010 at the Hospital de Clínicas José de San Martín, Buenos Aires, Argentina. All patients underwent radical retropubic prostatectomy (RRP) as their primary therapeutic strategy (date of RRP from December 1998 to July 2010). Patient recruitment, follow-up, and maintenance of updated medical records were performed by trained onco-urologists. All patients were Argentinian citizens and, by definition, Hispanic. Most of them had predominant Caucasian ancestry, although as reported for this population, some admixture of Amerindian and African ancestry is to be expected [23].

The study protocol was approved by the Institutional Ethical Committee and followed the Ethical Principles set forth by the Declaration of Helsinki. All patients who agreed to participate in the study signed a written informed consent form.

### 2.2. Genotyping

**Figure 1:**
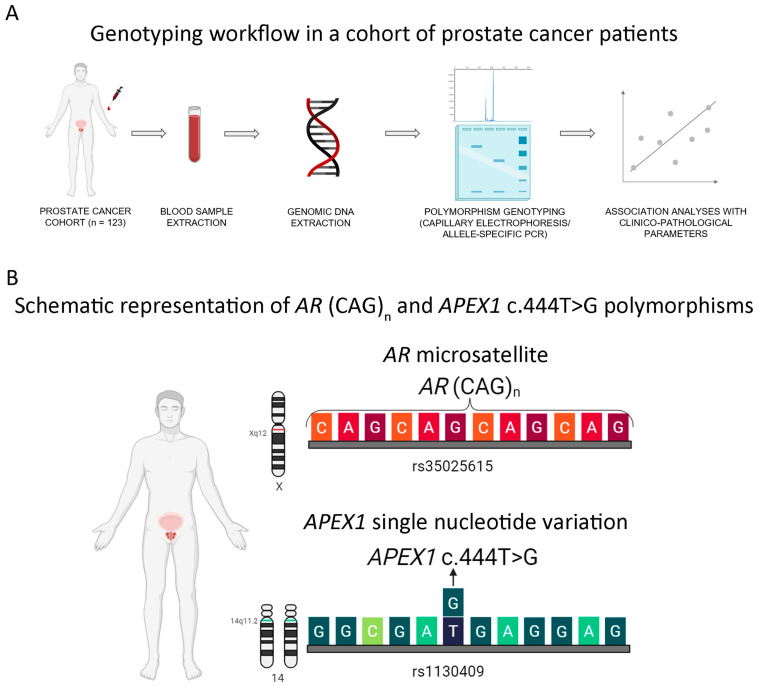
Genotyping workflow and polymorphism schematics. (**A**) The workflow for genotyping. Briefly, 123 Argentinian PCa patients (Hospital de Clínicas José de San Martin, Buenos Aires, Argentina) were recruited and peripheral blood samples were obtained. Germline DNA was extracted and *AR* (CAG)_n_ and *APEX1* c.444T>G were genotyped by capillary electrophoresis and allele-specific PCR, respectively. Finally, we performed association studies between the polymorphisms and clinicopathological features. (**B**) A schematic representation of the two polymorphisms studied: *AR* (CAG)_n_ microsatellite (rs3032358, upper panel) and *APEX1* c.444T>G (rs1130409, lower panel).

Germline DNA was extracted from peripheral blood during PCa follow-up using the CTAB method. We used PCR-based methods to genotype the *AR* (CAG)_n_ (rs3032358; NC_000023.11:g.67545317GCA[n]) and *APEX1* (rs1130409; NM_001641.4:c.444T>G; NP_001632.2:p.Asp148Glu) polymorphisms (Figure 1A,B). Of note, the current nomenclature for the *AR* microsatellite is (GCA)_n_; however, the vast majority of the literature refers to this polymorphism as (CAG)_n_ because it is the codon encoding for glutamine (Q). Therefore, we will use the more common (CAG)_n_ nomenclature to refer to this microsatellite to avoid confusion with other reports.

*AR* (CAG)_n_ was PCR-amplified with a labeled forward primer in a Veriti™ Thermal Cycler (Applied Biosystems™, Waltham, MA, USA) using the following reaction conditions: 1.5–2.5 ng/µL gDNA, 1× PCR reaction buffer (Productos Bio-Lógicos, Buenos Aires, Argentina), 200 µM dNTP (Genbiotech SRL, Buenos Aires, Argentina), 1.5 mM MgCl_2_ (Productos Bio-Lógicos, Buenos Aires, Argentina), 0.05 U/µL Taq polymerase (Productos Bio-Lógicos, Buenos Aires, Argentina), and 0.3 µM of each primer (forward: 5′-HEX-GAC CTA CCG AGG AGC TTT CC-3′ and reverse: 5′-AGA ACC ATC CTC ACC CTG CT-3′, IDT, Coralville, IA, USA). Thermocycling consisted of an initial denaturation step at 94 °C for 5 min, followed by 40 cycles of 94 °C for 30 s, 59 °C for 30 s, and 72 °C for 20 s, with a final extension step at 72 °C for 5 min. Amplicon sizes were determined by capillary electrophoresis (Applied Biosystems ABI 3730xl 96-capillary DNA Analyzer, Macrogen, Seoul, Republic of Korea) using the GeneScan™ 400HD ROX™ dye Size Standard and analyzed with Peak Scanner™ software v1.0 (Applied Biosystems™, Waltham, MA, USA). To determine the exact number of (CAG)_n_ repeats, four samples were sequenced using the Sanger method (Macrogen, Seoul, Republic of Korea) and then included as internal references for repeat numbers in all runs.

*APEX1* c.444T>G was genotyped using allele-specific PCR, as previously described [24]. Briefly, the PCR was performed using a mix of four primers: shared forward, 5′-CTT ACG GCA TAG GTG AGA CC-3′; shared reverse, 5′-GTC AAT TTC TTC ATG TGC CA-3′; T-specific forward, 5′-TCT GTT TCA TTT CTA TAG GCG AT-3′; and G-specific reverse, 5′-TCC TGA TCA TGC TCC TCC-3′. Amplicons were analyzed by 2% agarose gel electrophoresis stained with ethidium bromide.

Samples that failed to amplify or had unclear genotypes were re-processed once or twice as needed. Genotyping outputs were reviewed by two independent laboratory members, and 10–12% of the samples were randomly selected to be re-analyzed in a blinded manner for quality control. The results were included in the final analyses when there was 100% agreement between the two independent reviewers, and when there was 100% concordance between the samples and their blinded repeats.

Additionally, randomly selected samples were sequenced (Applied Biosystems ABI 3730xl 96-capillary DNA Analyzer, Macrogen, Seoul, Republic of Korea) to corroborate the genotypes and validate the experiments.

### 2.3. Statistical Analysis

Chi-square, Fisher’s exact, Kruskal–Wallis, or Wilcoxon rank-sum tests were performed as needed to analyze the association between genotypes and clinicopathological parameters, such as age at diagnosis, serum prostate-specific antigen (PSA) levels at diagnosis, family history of prostate/ovarian/breast cancer in first-degree relatives, surgical margin involvement, biochemical relapse, and ISUP (International Society of Urological Pathology) group.

The time to biochemical relapse was calculated from the date of RRP to the date of PSA rise above 0.2 ng/mL. To study the 6-year BRFS we considered the time to biochemical relapse or the time to last follow up (censored patients). Patients with follow-up times longer than 6 years or patients who had relapsed after that time were censored at 72 months. Kaplan–Meier plots were used to evaluate the association between genotypes and BRFS, and the comparison between groups was done using the log-rank test. Univariable and multivariable analyses were conducted using Cox proportional hazard models to study the association between the genotype and PCa biochemical relapse and to estimate hazard ratios (HR) and 95% confidence intervals (95% CI). Multivariable models included the following as covariates: margin involvement of the resected prostate, pathologic ISUP group, neoadjuvant therapy (androgen deprivation therapy or radiotherapy), pT stage, and serum PSA at diagnosis. All statistical analyses were performed in R [25] using the RStudio platform (RStudio, PBC, Boston, MA, USA, version 2024.04.1) [26].

### 2.4. Ethics Approval Statement

All procedures performed in studies involving human subjects were in accordance with the ethical standards of the institutional and/or national research committee and adhered to the 1964 Helsinki Declaration and its later amendments or comparable ethical standards.

## 3. Results

### 3.1. Clinicopathological Characteristics of the Studied Cohort

We studied a total of 123 Argentinian patients diagnosed with PCa. The patients’ clinicopathological characteristics are shown in Table 1. The median age at diagnosis was 64 years (range: 49–73), with a mean PSA = 9.85 ng/mL (median: 7.5; range: 0.77–84.1 ng/mL). Notably, 12 patients (9.76%) had serum PSA ≤ 4 ng/mL. Most patients were diagnosed with low- or favorable intermediate-risk tumors (ISUP 1 or 2; 78.87%). The majority of the resected prostates had tumor-free margins (76.67%), and 49.14% of patients were diagnosed with pT2-stage tumors. A total of 27 patients (25%) underwent biochemical relapse, with a median time to relapse of 31 months (range: 8–129 months). The overall median follow-up time was 83 months (range: 14–152 months), with a median follow-up time of 86.5 months for patients who had relapsed (range: 27–149 months) and of 80.5 months for recurrence-free patients (range: 14–152 months); this difference was not significant (*p* = 1). The median time to biochemical relapse for the entire cohort was 31 months (range: 8–129 months).

We studied the association between the aforementioned clinicopathological variables and PCa recurrence. Among these clinicopathological parameters, only ISUP grade and surgical margin involvement were significantly associated with the risk of biochemical relapse (log-rank *p* = 0.014 and log-rank *p* = 0.012, respectively; Table 1). We did not observe any association with the risk of biochemical relapse for the other three variables (*p*-T, neoadjuvant therapy, and serum PSA at diagnosis). Overall, our cohort followed the current clinical criteria used to evaluate the risk of PCa biochemical relapse.

### 3.2. Association Between AR (CAG)_n_ and PCa

Genotyping of *AR* (CAG)_n_ was successful in 111/123 patients (90.24%); the remaining 12 patients were considered failures after repeating the PCR three times. Some reports have shown *AR* (CAG)_n_ expansions with alleles having more than 38 repeats and, although the expansion of this microsatellite is usually associated with neurological disorders such as Spinal and Bulbar Muscular Atrophy (SBMA, also known as Kennedy’s disease), a recent study that analyzed 74,277 individuals (males and females) found a higher-than-expected *AR* (CAG)_n_ expansion in the general population (non-SBMA) [27]. Therefore, even though the most likely explanation for PCR failure is low DNA quality, we cannot rule out the idea that it was also due to microsatellite expansion exceeding the PCR amplification range.

**Figure 2:**
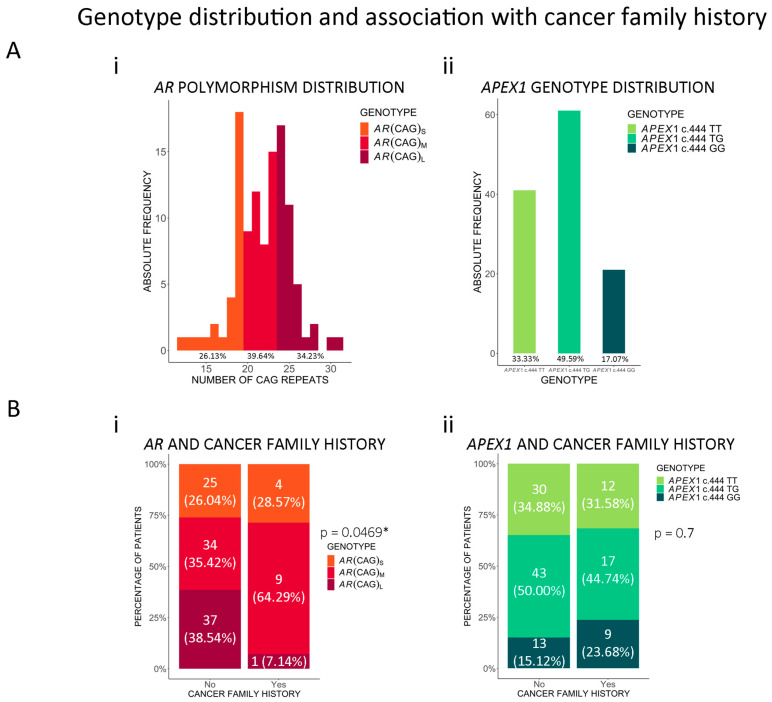
*AR* (CAG)_n_ and *APEX1* c.444T>G genotype distribution and association with cancer family history. (**A**) (i) A histogram depicting the continuous distribution and absolute frequencies of (CAG)_n_ length in *AR*. Patients were classified according to the length of the polymorphism in short-length (*AR* (CAG)_S_: <20 repeats, n = 29, orange), medium-length (*AR* (CAG)_M_: 20–23 repeats, n = 44, red), and long-length (*AR* (CAG)_L_: >23 repeats, n = 38, dark red) alleles. (ii) A bar plot showing the frequency of *APEX1* c.444TT (n = 41, green), *APEX1* c.444TG (n = 61, light green), and *APEX1* c.444GG (n = 21, dark green) genotypes. (**B**) (i) Analysis of AR (CAG)_n_ distribution between patients with and without a family history of breast/ovarian cancer. (ii) Analysis of *APEX1* c.444T>G frequencies in patients with and without a family history of prostate/breast/ovarian cancer. Statistical significance was assessed by the chi-square test. * *p* < 0.05.

Because *AR* is located on Xq12, men are hemizygous and, therefore, have only one allele. The number of (CAG)_n_ repeats in our cohort ranged from 12 to 31, with a median of 22 repeats (Figure 2A(i)), and 19 and 24 repeats were the most frequent among the alleles. The distribution of each clinicopathological variable was similar to that described by others for Hispanic and Latin American populations [10,28,29] and is shown in Supplementary Table S1. We categorized the patients according to the number of (CAG)_n_ repeats as follows: *AR* (CAG)_S_ (short-length alleles: <20 repeats), *AR* (CAG)_M_ (medium-length alleles: 20–23 repeats), and *AR* (CAG)_L_ (long-length alleles: >23 repeats), with frequencies of 26.13%, 39.64%, and 34.23%, respectively (Figure 2A(i)). This categorization was defined based on the tertile distribution, with <20 and >23 repeats chosen as the cut-off points. These cut-off points are in agreement with the literature, although they vary across studies [10,28,29].

We then studied the association between the (CAG)_n_ groups and PCa clinicopathological characteristics in univariable and multivariable analyses. We found significant differences in the distribution of *AR* (CAG)_n_ genotypes between patients with and without a family history of breast/ovarian cancer in first-degree relatives (*p* = 0.047, Figure 2B(i)). Prostate cancer family history was not included in this analysis because of the maternal inheritance of the X chromosome, which discards a genetic component of first-grade relatives with prostate cancer that could be associated with this polymorphism. We observed that (CAG)_L_ was the most frequent allele among PCa patients without a family history of cancer (38.54% vs. 7.14% in patients with a family history of cancer), and the frequency of (CAG)_M_ was higher in patients with a family history of cancer (64.29% vs. 35.42% in patients without cancer family history). These findings suggest that the *AR* (CAG)_M_ genotype might be associated with a higher genetic predisposition to PCa.

**Figure 3:**
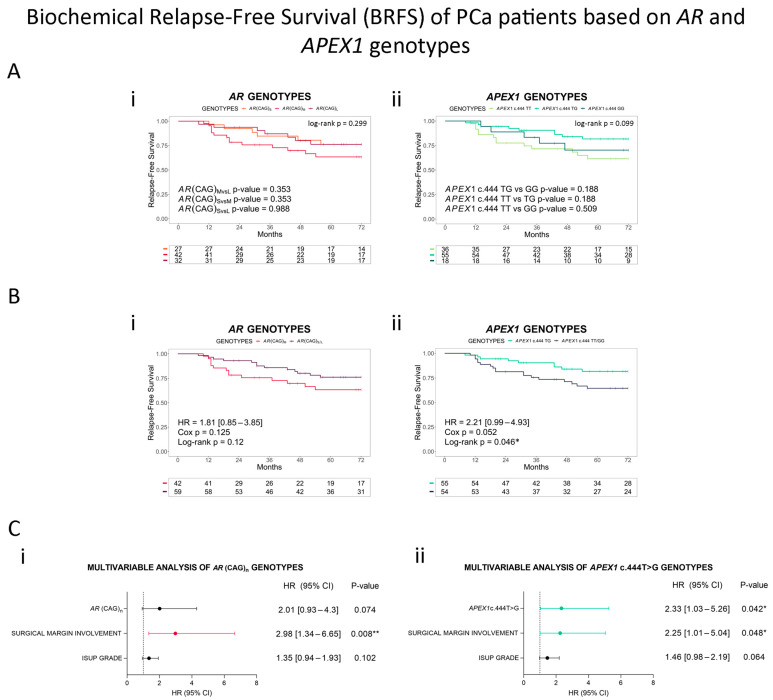
Biochemical Relapse-Free Survival (BRFS) in Argentinian PCa patients based on *AR* and *APEX1* genotypes. (**A**) Kaplan–Meier curves for BRFS in Argentinian PCa patients stratified by (i) *AR* (CAG)_n_ or (ii) *APEX1* c.444T>G (p.Asp148Glu). (**B**) Kaplan–Meier curves for BRFS in Argentinian PCa patients stratified by grouped genotypes (i) *AR* (CAG)_S/L_ vs. *AR* (CAG)_M_ (reference genotype) or (ii) *APEX1* c.444TT/GG vs. *APEX1* c.444TG (reference genotype). (**C**) Forest plots depicting hazard ratio (HR) estimation in multivariable analyses including histopathological parameters (surgical margin involvement and ISUP grade) and (i) *AR* (CAG)_n_ or (ii) *APEX1* c.444T>G as covariates. Adjusted *p*-values were calculated by Cox regression. HR (95% CI) = hazard ratio (95% confidence interval). Cox *p* = *p*-value of Cox proportional hazards model. Statistical significance was set at log-rank *p*-value/Cox *p* < 0.05. * Cox *p* < 0.05; ** Cox *p* < 0.01.

We also performed a survival analysis to study the association with BRFS. When we analyzed the three *AR* genotypes independently, we did not find a significant difference (log-rank *p* = 0.299, Figure 3A(i)). Particularly, since we observed similar survival between (CAG)_S_ and (CAG)_L_ alleles (Figure 3A(i)), we grouped them into a new category, (CAG)_S/L_. Although this new grouping might seem unintuitive at first glance, it is supported by other reports suggesting that both short and long alleles reduce AR activity by interfering with coregulators’ interactions [28,29,30]. When we analyzed the 6-year BRFS between (CAG)_M_ and (CAG)_S/L_, we found that the patients with a medium-length allele had a reduced BRFS and a nearly 2-fold risk of biochemical relapse, although this was not significant (*p* = 0.125, Figure 3B(i)). Finally, we constructed a multivariable predictive model including the *AR* genotype and the clinicopathological variables associated with relapse in our cohort (ISUP grade and surgical margin involvement) as covariates. We found a marginally significant increased risk of biochemical relapse in patients with medium-length alleles compared with patients that had short-/long-length alleles (HR = 2.01, 95% CI = 0.93–4.30, *p* = 0.074, Figure 3C(i)).

**Figure 4:**
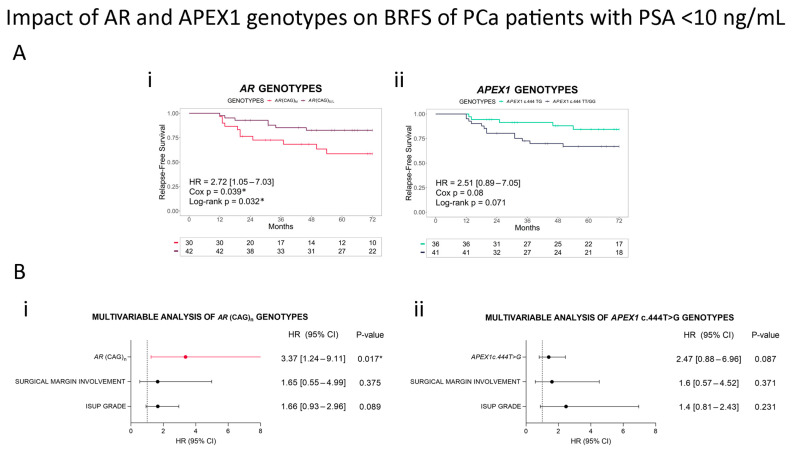
Impact of *AR* and *APEX1* genotypes on BRFS in Argentinian PCa patients with serum PSA<10 ng/mL. (**A**) Kaplan–Meier curves for biochemical relapse-free survival (BRFS) in Argentinian PCa patients with serum PSA < 10 ng/mL, classified by (i) *AR* (CAG)_S/L_ vs. *AR* (CAG)_M_ (reference genotype) or (ii) *APEX1* c.444TT/GG vs. *APEX1* c.444TG (reference genotype). (**B**) Forest plots depicting hazard ratio (HR) estimation in multivariable analyses including histopathological parameters (surgical margin involvement and ISUP grade) and (i) *AR* (CAG)_n_ or (ii) *APEX1* c.444T>G. Adjusted *p*-values were calculated by Cox regression. HR (95% CI) = hazard ratio (95% confidence interval). Cox *p* = *p*-value of Cox proportional hazards model. Statistical significance was set at log-rank *p*-value/Cox *p* < 0.05. * Cox *p* < 0.05.

Given PCa’s heterogeneity and the higher uncertainty of disease outcome for patients with PSA < 10 ng/mL, we furthered our analysis on patients within this group. The (CAG)_M_ allele significantly reduced the 6-year BRFS (*p* = 0.032), with a 2.7-fold risk of relapse (HR = 2.72, 95% CI = 1.05–7.03, *p* = 0.039, Figure 4A(i)). The multivariable analysis including ISUP grades and surgical margin involvement showed that *AR* (CAG)_n_ was an independent risk factor, with an over 3-fold risk of relapse for patients with medium-length alleles (HR = 3.37, 95% CI = 1.24–9.11, *p* = 0.017, Figure 4B(i)).

### 3.3. Association Between APEX1 c.444T>G (p.Asp148Glu) and PCa

Genotyping of *APEX1* c.444T>G was successful in 123/123 patients (100%). Genotype frequencies were 33.33% c.444TT, 49.59% c.444TG, and 17.07% c.444GG (Figure 2A(ii)), and allele frequencies were 58% c.444T and 42% c.444G. Genotypes were in Hardy–Weinberg Equilibrium and the distribution within each clinicopathological variable is shown in Supplementary Table S1. These frequencies were similar to those described for Global and Latin American populations in dbSNP [31]. We did not observe significant associations between *APEX1* c.444T>G genotypes and clinicopathological features (Figure 2B(ii) and Supplementary Table S1).

When we analyzed the 6-year BRFS, we found marginally significant differences between the groups (log-rank *p* = 0.099, Figure 3A(ii)). Because we observed that heterozygous patients had better survival rates, although the difference was non-significant, we merged homozygous T and G patients into one group. This new grouping is supported by other studies where the heterozygous genotypic model has been associated with disease development and outcome [17,32]. We found that c.444TG patients had a significantly better 6-year BRFS compared to c.444TT/GG patients, with a 2-fold higher risk of relapse in homozygous TT/GG patients compared with heterozygous men (Figure 3B(ii)). The multivariable model including the ISUP grade and surgical margin involvement as covariates showed that *APEX1* c.444T>G is an independent risk factor (HR = 2.33, 95% CI = 1.03–5.26, *p* = 0.042, Figure 3C(ii)). We did not find a significant association when we considered only patients with PSA <10 ng/mL; however, *APEX1* SNV is a marginally significant biomarker of PCa relapse (Figure 4A(ii),B(ii)).

### 3.4. AR (CAG)_n_ and APEX1 c.444T>G (p.Asp148Glu) Interaction as a Predictor of PCa Relapse

**Figure 5:**
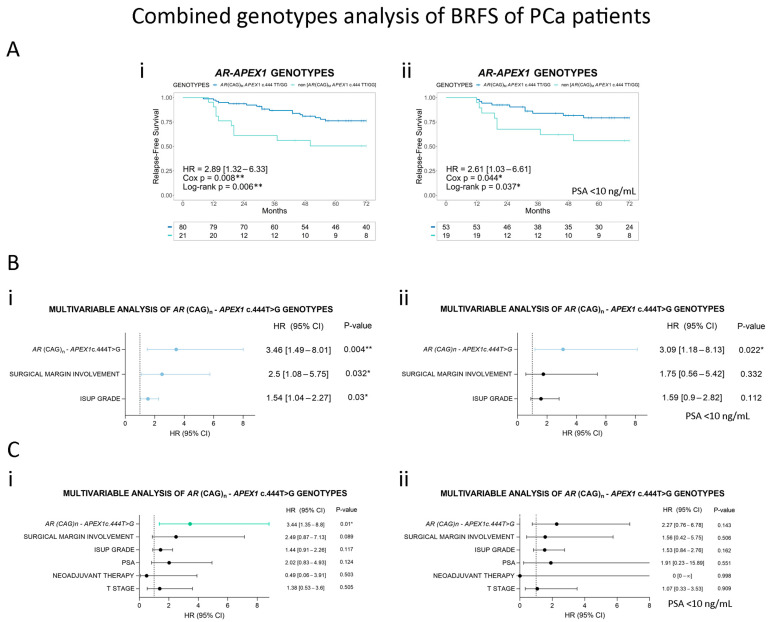
Combined genotypes analysis of BRFS in Argentinian PCa patients. (**A**) Kaplan–Meier curves for biochemical relapse-free survival (BRFS) in (i) the total cohort of Argentinian PCa patients and (ii) the patients with PSA < 10 ng/mL, grouped by *AR* (CAG)_n_ + *APEX1* c.444T>G. (**B**) Forest plots of hazard ratio (HR) estimation in multivariable analyses including histopathological parameters (surgical margin involvement and ISUP grade) and *AR* (CAG)_n_ + *APEX1* c.444T>G genotype as covariates for (i) the total cohort and (ii) the PSA < 10 ng/mL subgroup. (**C**) Forest plots of hazard ratio (HR) estimation in multivariable analyses including surgical margin involvement, ISUP grade, serum PSA, neoadjuvant therapy, and pT stage as covariates for (i) the total cohort and (ii) the PSA < 10 ng/mL subgroup. In all analyses, the *AR* (CAG)_M_ + *APEX1* c.444TT/GG genotype was considered as the reference group. HR (95% CI) = hazard ratio (95% confidence interval). Cox *p* = *p*-value of Cox proportional hazards model. Statistical significance was set at log-rank *p*-value/Cox *p* < 0.05. * Cox *p* < 0.05; ** Cox *p* < 0.01.

Finally, we evaluated whether the combination of *AR* (CAG)_n_ and *APEX1* c.444T>G genotypes has a combinatorial association with the risk of PCa relapse. We considered the combination of *AR* (CAG)_M_ + *APEX1* c.444TT/GG as a risk genotype and compared it to *AR* (CAG)_S/L_ + *APEX1* c.444TG (Supplementary Table S2). We found that PCa patients with the *AR* (CAG)_M_ + *APEX1* c.444TT/GG combination had an over 2.5-fold increased risk of undergoing biochemical relapse within 6 years compared to patients with other genotypes. This association was significant for both the entire cohort (HR = 2.89, 95% CI = 1.32–6.33, *p* = 0.008, Figure 5A(i)) and the subgroup of patients with PSA < 10 ng/mL (HR = 2.61, 95% CI = 1.03–6.61, *p* = 0.044, Figure 5A(ii)) in the univariable analysis. The multivariable analysis including the ISUP grade and surgical margin involvement confirmed these results and showed an over 3-fold significant increased risk for both groups (Figure 5B(i,ii)), suggesting the independent role of the combination of *AR* (CAG)_n_ and *APEX1* c.444T>G in predicting PCa relapse. To further confirm these findings in a more complex predictive model, we evaluated a multivariable model including the combined genotype, ISUP grade, surgical margin involvement, PSA at diagnosis, neoadjuvant therapy, and pT-stage as covariates. We found that the combined genotype was the only independent significant risk factor associated with biochemical relapse (HR = 3.44, 95% CI = 1.35–8.80, *p* = 0.010, Figure 5C(i)). The significant associations of ISUP and margin involvement were lost in this multivariable model (Figure 5C(i)). Figure 5C(ii) shows the latter multivariable model for the subgroup of patients with PSA < 10 ng/mL, where no significant associations were observed.

Overall, we found that *AR* (CAG)_n_ and *APEX1* c.444T>G polymorphisms are genetic predisposition factors for PCa and that they might modulate the disease outcome in the context of other known risk factors. These results highlight the potential use of these polymorphisms as prognostic biomarkers in PCa.

## 4. Discussion

In this paper, we present the first evidence of an association between *AR* (CAG)_n_ and *APEX1* c.444T>G polymorphisms and the risk of biochemical recurrence in Argentine patients with prostate cancer (PCa). Our results show that these polymorphisms are linked to reduced biochemical relapse-free survival, indicating a possible inherited genetic predisposition to PCa progression. Globally, PCa is the second most frequently diagnosed cancer and the fifth leading cause of cancer-related mortality in men [1]. In Argentina, it is the most common malignancy and ranks third among cancer-related deaths in men [1]. Predicting the clinical course of localized PCa remains a significant challenge, as patients with similar tumor characteristics can exhibit widely divergent outcomes. Despite advances in diagnostic and prognostic methodologies, the inherent heterogeneity of PCa continues to limit the ability of current staging systems to accurately predict disease progression [33,34]. Hence, incorporating germline genetic variants into predictive models represents a promising avenue for improving prognostic precision and aiding clinicians in making precise therapeutic decisions.

The *AR* gene contains a (CAG)_n_ codon repeat polymorphism in the first exon, which encodes a polyglutamine (polyQ) tract in the N-terminal transactivation domain. It has been extensively reported that the length of this polyQ tract is inversely correlated with both basal and ligand-induced AR transactivation activity. Additionally, the polyQ tract’s length has been linked to various traits and diseases in both males [35,36,37,38,39,40,41] and females [42,43,44]. However, the effect of this polymorphism on disease development and progression remains unclear and controversial, with some authors suggesting region- and ethnicity-specific effects [11,42,43].

In this study, we found that PCa patients with medium-length (CAG)_n_ alleles, (CAG)_20–23_, had worse biochemical relapse-free survival rates compared to those with short- or long-length alleles, (CAG)_<20_ or (CAG)_>23_. Although this grouping may initially seem arbitrary, a meta-analysis reported similar findings: men with (CAG)_21–24_ had a higher risk of developing testicular germ cell tumors (OR = 1.54, 95% CI = 1.18–2.02, *p* = 0.002) compared to men with (CAG)_<21_ or (CAG)_>24_ alleles [40]. Another meta-analysis reported similar results, showing that men with (CAG)_<21_ or (CAG)_>25_ alleles had a lower risk of testicular cancer (OR = 0.81, 95% CI = 0.68–0.96, *p* = 0.017) compared to men with (CAG)_21-25_ alleles in a population-based analysis [41].

Additionally, an in vivo study using humanized AR transgenic adenocarcinoma of mouse prostate (TRAMP) mice, created by recombining a human *AR* (CAG)_(12, 21, or 48)_ DNA fragment with the mouse *Ar* gene, demonstrated that longer microsatellites provided an effect that was more protective against PCa development (PCa incidence: 85%, 52%, and 28%, respectively) [30]. Interestingly, mice with (CAG)_21_ showed higher PCa-related mortality compared to mice with (CAG)_(12 or 48)_ [30]. These findings are also consistent with in vitro studies suggesting that both short and long alleles reduce AR activity by disrupting interactions with coregulators [28]. Altogether, these results suggest that, although shorter *AR* (CAG)_n_ is associated with an increased risk of developing PCa, medium-length alleles may be a risk factor for disease progression once the tumor has developed. Moreover, the significant association between medium-length *AR* (CAG)_n_ and a family history of breast and ovarian cancer suggests that this polymorphism may contribute to the genetic burden in familial cancer clustering.

APEX1 is an enzyme involved in the DNA base excision repair pathway [14,45,46,47] that is recruited to androgen response element regions to help modulate the transcription of AR target genes [15]. The *APEX1* SNV c.444T>G (p.Asp148Glu) has been reported to be innocuous with respect to DNA binding activity. In line with this evidence, association meta-analyses across all genetic models (dominant, recessive, codominant, additive, and allelic) have either not supported this SNV as a risk factor for various types of cancer (including PCa) or have found only weak associations in certain subgroups or populations [46,48,49,50].

Herein, we observed an increased risk of PCa biochemical relapse in patients with the c.444TG genotype compared to homozygous patients. This codominant genetic model is supported by other studies that have reported a significantly higher risk of developing PCa in Brazilian populations only among heterozygous patients (OR_TGvsTT_ = 1.64, 95% CI = 1.06–2.25, *p* = 0.03; OR_GGvsTT_ = 3.10, 95% CI = 0.59–16.67, *p* = 0.30) [17]. Moreover, one meta-analysis that included 58 articles and 48,903 participants with various types of cancer made an overall estimate of OR = 1.08 (95% CI = 1.01–1.16; *p* = 0.026) specifically for the heterozygous genetic model [32].

Finally, to test the hypothesis of an epistatic effect between these two polymorphisms that impacts PCa biochemical relapse risk, we combined both genotypes and performed a biochemical relapse-free survival analysis. We observed that patients with both risk genotypes (*AR* (CAG)_M_ + *APEX1* c.444TT/GG) had the shortest median time to relapse, emphasizing the potential of using these combined markers to identify high-risk patients. This is particularly relevant for patients with PSA < 10 ng/mL, where traditional markers may not sufficiently predict outcomes. Our findings suggest that incorporating these genetic markers into prognostic models could enhance risk stratification and guide more personalized treatment approaches.

To determine the specific contribution of *AR* and *APEX1* polymorphisms to the prognosis of PCa, it is essential to evaluate their effects independently of other known clinical factors. This analysis helps to establish the robustness and reliability of these genetic markers in predicting disease outcomes. Our multivariable analysis confirmed that *AR* and *APEX1* polymorphisms are independent predictors of biochemical relapse, even when accounting for other prognostic histological variables such as surgical margin involvement and pathologic ISUP grade. This comprehensive analysis underscores the prognostic value of *AR* and *APEX1* polymorphisms in assessing the risk of biochemical relapse in PCa patients and highlights their clinical utility.

## 5. Conclusions

In conclusion, this study demonstrates the significant prognostic value of germline *AR* (CAG)_n_ and *APEX1* p.Asp148Glu polymorphisms in Argentinian PCa patients. Their assessment through simple blood tests might provide valuable prognostic information, aiding in precise treatments. Further research in larger and more diverse cohorts is warranted to validate these findings and explore their integration into clinical practice.

### Limitations

Despite the strengths of our study, some limitations should be acknowledged. First, the sample size, although sufficient for initial analysis, may not capture the full genetic diversity of the Argentinian population. Larger prospective and multi-center studies are needed to validate our findings. Second, cancer risk results from a complex interplay of genetic predispositions, environmental exposures, age, and sex—factors that were not fully accounted for in this study. Third, there are other frequent polymorphisms in *AR* and *APEX1* that were not studied here, and they may also modulate the risk of PCa relapse.

## Figures and Tables

**Table 1:** The clinicopathological characteristics of the 123 Argentinian PCa patients included in the study cohort.

Characteristics	n = 123	Association with 6-Year BRFS (Log-Rank *p*)
Age at diagnosis ^1^	64 (49–73)	0.475
Cancer family history ^2^		0.345
No	85 (69.67%)	
Yes	37 (30.33%)	
Missing	1	
Surgical margin involvement ^2^		0.012 *
No	92 (76.67%)	
Yes	28 (23.33%)	
Missing	3	
PSA (ng/mL) ^1,2^	7.51 (0.77–84.10)	0.7
≤4	12 (9.76%)	
4–10	68 (55.28%)	
≥10	43 (34.96%)	
Biochemical relapse ^2^		-
No	82 (75.45%)	
Yes	27 (24.55%)	
Missing	14	
Follow-up time (months) ^1^		-
Without BCR	80.5 (14–152)	
With BCR	86.5(27–149)	
Time to biochemical relapse (months) ^1^	31 (8–129)	-
ISUP grade ^2^		0.014 *
1	58 (47.16%)	
2	39 (31.71%)	
3	17 (13.82%)	
4	8 (6.5%)	
5	1 (0.81%)	
pT stage ^2^		0.082
2	57 (49.14%)	
3a	53 (45.69%)	
3b	6 (5.17%)	
Missing	7	
Neoadjuvant therapy ^2^		0.4
No	114 (92.68%)	
Yes	9 (7.32%)	

^1^ Median (range). ^2^ n (%). * Log-Rank *p* < 0.05.

## Data Availability

The data will be made available by the authors upon reasonable request.

## Supplementary Materials

The following supporting information can be downloaded at: https://www.mdpi.com/article/10.3390/cancers16223815/s1, Supplementary Tables: Supplementary Table S1—Clinicopathological characteristics stratified by AR and APEX1 genotypes; Supplementary Table S2—Clinicopathological characteristics stratified by combined AR and APEX1 genotypes.
