# Supplementary material for: AR (CAG)n Microsatellite and APEX1 c.444T>G (p.Asp148Glu) Polymorphisms as Independent Prognostic Biomarkers in Prostate Cancer: Insights from an Argentinian Cohort
Source: Cancers (Basel). 2024 Nov 13;16(22):3815. doi: 10.3390/cancers16223815 (PMC11592882; doi:10.3390/cancers16223815)
Supplement: Supplementary file 1 [file cancers-16-03815-s001.zip › cancers-3279035-supplementary.pdf]

Supplementary Table S1: Clinicopathological characteristics stratified by *AR* and *APEX1* genotypes.

	***AR* (CAG)$_n$**				***APEX1* c.444T>G**			
**Characteristics**	**S (n = 29)**	**M (n = 44)**	**L (n = 38)**	**p-value[3]**	**TT (n = 41)**	**TG (n = 61)**	**GG (n = 21)**	**p-value[3]**
**Age at diagnosis[1]**	65 (50-71)	64.0 (49-72)	63.5 (55-73)	0.37	64.0 (49-73)	64 (49-72)	63 (53-70)	0.51
**Cancer family history[2]**				0.047*				0.7
No	25 (86.21%)	34 (79.07%)	37 (97.37%)		29 (70.73%)	43 (71.67%)	13 (61.9%)	
Yes	4 (13.79%)	9 (20.93%)	1 (2.63%)		12 (29.27%)	17 (28.33%)	8 (38.1%)	
Missing	0	1	0		0	1	0	
**Surgical margin involvement[2]**				0.7				0.7
No	23 (82.14%)	31 (73.81%)	30 (78.95%)		29 (72.5%)	47 (79.66%)	16 (76.19%)	
Yes	5 (17.86%)	11 (26.19%)	8 (21.05%)		11 (27.5%)	12 (20.34%)	5 (23.81%)	
Missing	1	2	0		1	2	0	
**PSA (ng/mL)[1,2]**	7.1 (0.77-28.9)	6.67 (0.90-84.1)	7.80 (3.06-20.3)	0.6	7.80 (0.77-28.9)	6.9 (2.66-27)	8.4 (4.52-84.1)	0.3
≤ 4	5 (17.24%)	3 (6.82%)	4 (10.53%)		6 (14.63%)	6 (9.84%)	0 (0%)	
4-10	13 (44.83%)	27 (61.36%)	21 (55.26%)		22 (53.66%)	31 (50.82%)	15 (71.43%)	
≥ 10	11 (37.93%)	14 (31.82%)	13 (34.21%)		13 (31.71%)	24 (39.34%)	6 (28.57%)	
**Biochemical relapse[2]**				0.5				0.093
No	21 (77.78%)	29 (67.44%)	24 (77.42%)		23 (63.89%)	45 (83.33%)	14 (73.68%)	
Yes	6 (22.22%)	14 (32.56%)	7 (22.58%)		13 (36.11%)	9 (16.67%)	5 (26.32%)	
Missing	2	1	7		5	7	2	
**Follow-up time (months)[1]**								
Without BCR	81 (14-122)	73 (19-137)	82.5 (22-152)	0.72	83 (19-152)	75 (14-137)	82 (35-114)	0.69
With BCR	88 (65-112)	83 (27-119)	101 (64-149)	0.23	83 (27-119)	95.5 (34-146)	88 (51-149)	0.48
**Time to biochemical relapse (months)[1]**	31 (12-73)	20 (10-80)	44.5 (8-129)	0.17	20 (10-56)	43 (8-120)	37 (14-129)	0.14
**ISUP grade[2]**				0.3				0.6
1	14 (48.28%)	22 (50%)	20 (52.63%)		22 (53.66%)	26(42.62%)	10 (47.62%)	
2	12 (41.38%)	9 (20.45%)	11 (28.95%)		10 (24.39%)	23 (37.7%)	6 (28.57%)	
3	1 (3.45%)	10 (22.73%)	5 (13.16%)		7 (17.07%)	7 (11.48%)	3 (14.29%)	
4	2 (6.89%)	3 (6.82%)	2 (5.26%)		1 (2.44%)	5 (8.2%)	2 (9.52%)	
5	0 (0%)	0 (0%)	0 (0%)		1 (2.44%)	0 (0%)	0 (0%)	
**pT Stage[2]**				0.5				0.5
2	13 (46.43%)	19 (47.5%)	20 (54.05%)		20 (54.05%)	26 (44.83%)	11 (52.38%)	
3a	13 (46.43%)	20 (50%)	15 (40.54%)		15 (40.54%)	30 (51.72%)	8 (38.1%)	
3b	2 (7.14%)	1 (2.5%)	2 (5.41)		2 (5.41%)	2 (3.45%)	2 (9.52%)	
Missing	1	4	1		4	3	0	
**Neoadjuvant Therapy[2]**				0.3				0.8
No	28 (96.55%)	42 (95.45%)	33 (86.84%)		37 (90.24%)	57 (93.44%)	20 (95.24%)	
Yes	1 (3.45%)	2 (4.55%)	5 (13.16%)		4 (9.76%)	4 (6.56%)	1 (4.76%)	

[1] Median (Range)

[2] n (%)

[3] Kruskal-Wallis/Wilcoxon rank sum test; Pearson's Chi squared test; Fisher's

*p <0.05

Supplementary Table S2. Clinicopathological characteristics stratified by the combined *AR* and *APEX1* genotypes.

Characteristics	TT/GG M (n = 23)	Non TT/GG M (n = 88)	p-value[3]
**Age at diagnosis[1]**	64 (49-71)	64 (50-73)	0.95
**Cancer family history[2]**			0.036*
No	12 (52.17%)	65 (74.71%)	
Yes	11 (47.83%)	22 (25.29%)	
Missing	0	1	
**Surgical margin involvement[2]**			0.1
No	15 (65.22%)	69 (81.18%)	
Yes	8 (34.78%)	16 (18.82%)	
Missing	0	3	
**PSA (ng/mL)[1,2]**	8.2 (3.15-84.1)	7 (0.77-28.9)	0.11
≤ 4	2 (10.53%)	10 (11.36%)	
4-10	17 (89.47%)	44 (50%)	
≥ 10	4	34 (38.64%)	
**Biochemical relapse[2]**			0.023*
No	12 (54.55%)	62 (78.48%)	
Yes	10 (45.45%)	17 (21.52%)	
Missing	1	9	
**Follow-up time (months)[1]**			
Without BCR	82.5 (19-111)	80 (14-152)	0.86
With BCR	83 (27-119)	88 (34-149)	0.43
**Time to biochemical relapse (months)[1]**	19 (10-80)	43 (8-129)	0.06
**ISUP grade[2]**			0.056
1	15 (65.22%)	41 (46.59%)	
2	2 (8.69%)	30 (34.09%)	
3	5 (21.74%)	11 (12.5%)	
4	1 (4.35%)	6 (6.82%)	
5	0	0	
**pT Stage[2]**			0.7
2	10 (47.62%)	42 (50%)	
3a	10 (47.62%)	38 (45.24%)	
3b	1 (4.76%)	4 (4.76%)	
Missing	2	4	
**Neoadjuvant Therapy[2]**			> 0.9
No	22 (95.65%)	81 (92.05%)	
Yes	1 (4.35%)	7 (7.95%)	

[1] Median (Range)

[2] n (%)

[3] Kruskal-Wallis/Wilcoxon rank sum test; Pearson's Chi squared test; Fisher's

*p <0.05
